# Supplementary material for: Collaborative intelligence in AI: Evaluating the performance of a council of AIs on the USMLE
Source: PLOS Digit Health. 2025 Oct 9;4(10):e0000787. doi: 10.1371/journal.pdig.0000787 (PMC12510544; doi:10.1371/journal.pdig.0000787)
Supplement: S2 File — (ZIP) [file pdig.0000787.s002.zip › S2 Data Raw data files containing unprocessed question inputs and LLM outputs including transcripts of deliberations/STEP3_Question-44-1_Type-MC-NJ.docx]

**Facilitator** **[Round 1]:** A 70-year-old woman comes to the office for an annual health maintenance examination. She describes a 1-year history of slowly progressive fatigue, diffuse muscle aches, and generalized pain. Medical history is significant for osteoporosis diagnosed 5 years ago. Medications include alendronate and calcium supplementation. Vital signs are normal. Physical examination is unremarkable for her age, with no point tenderness on palpation. Results of complete blood count, serum electrolyte and thyroid-stimulating hormone concentrations, liver function tests, and erythrocyte sedimentation rate are all within the reference ranges. Screening mammography and colonoscopy done 1 year ago disclosed no abnormalities. Which of the following is the most appropriate next step in evaluation? (A) DEXA scan (B) Electromyography and nerve conduction studies (C) MRI of the cervical spine (D) Serum antinuclear antibody assay (E) Serum 25-hydroxyvitamin D assay (F) No additional evaluation is indicated

**AI_B:** RESTATING THE QUESTION: A 70-year-old woman with a history of osteoporosis and generalized pain complaints, whose test results are all within the reference ranges, is in need of the next appropriate step for evaluation. The possible steps include: a bone density test known as DEXA scan, an electromyography and nerve conduction studies to assess her nerves and muscle condition, an MRI of the cervical spine, a serum antinuclear antibody assay to check for autoimmune diseases, a serum 25-hydroxyvitamin D assay to measure her vitamin D levels, or a suggestion that no further evaluation is necessary. PLANNING: Assumptions drawn from her complaints could include a potential deficiency or imbalance (as fatigue and muscle aches could imply), an issue with nerve conduction (given the muscle aches), a spinal problem, an undiagnosed autoimmune disease or no clinically evident issue. Each assumption could then be investigated by the corresponding test, with potential clinical priority given to vitamin D deficiency, as it can cause muscle ache and is common in osteoporosis patients. CLINICAL REASONING: Fatigue, muscle aches, and generalized pain might stem from different sources. Given the patient's demographic, history of osteoporosis, and the use of calcium supplement, it would be rational to suspect a Vitamin D deficiency. Vitamin D plays a crucial role in the absorption of calcium and it is also important for bone health. Hypovitaminosis D often presents with generalized complaints such as muscle aches and general fatigue. Furthermore, it is very common in the elderly (especially those with osteoporosis) due to decreased dietary intake, lack of sun exposure, and impaired activation in the kidneys. CONCLUDING: Given her age, history of osteoporosis, medication use, and presenting symptoms of fatigue and muscle aches, a preferred way of investigating her condition would be to assess her vitamin D levels. A deficiency could lead to the aforementioned symptoms, and its prevalence in the elderly with osteoporosis is quite high; indeed, failing to address it could lead to further bone weakening. RESPONSE: Therefore, the next appropriate step in this woman's evaluation would be (E) Serum 25-hydroxyvitamin D assay.

**AI_A:** 1) RESTATING THE QUESTION: An elderly woman presents with a year-long history of vague symptoms including fatigue, widespread muscle aches, and overall discomfort. She has a previous diagnosis of osteoporosis and is currently taking alendronate and calcium supplements. Her current medical evaluation, including lab work and imaging, shows no apparent cause of her symptoms. We need to decide the most clinically appropriate next course of investigation for her. 2) PLANNING: The initial assumption may be that her symptoms are associated with her age. However, given their progressive nature and duration, an alternate hypothesis could be an underlying condition not yet picked up in her screening assessments. Conditions like vitamin D deficiency, systemic autoimmune disorders, or neurological disorders may present similarly and might not be detected in standard screenings, therefore should be further investigated. 3) CLINICAL REASONING: Considering her age, medical history, and current medications, her symptoms of aches and generalized pain could be associated with osteoporosis. However, her current treatment with alendronate and calcium supplementations should ideally be managing this, and generalized pain/fatigue is not a typical presentation for osteoporosis. Additionally, she has no point tenderness on examination, which is expected in osteoporosis if the pain was due to a fracture. Hence, osteoporosis is unlikely to be the cause of her symptoms. Other options such as systemic autoimmune disorders or neurological disorders can present with generalized pain and fatigue but are unlikely given the normal blood work and the absence of specific clinical signs on examination. Vitamin D deficiency, on the other hand, could present with diffuse muscle aches, fatigue, and generalized pain. Given her age, it is quite possible as elderly individuals are at a high risk for vitamin D deficiency due to decreased dietary intake, reduced sunlight exposure, and diminished capacity for cutaneous synthesis of Vitamin D. 4) CONCLUDING: With the information provided and the clinical reasoning process adopted, it is reasonable to think that the woman's symptoms could be due to Vitamin D deficiency. 5) RESPONSE: The most appropriate next step in evaluation would be (E) Serum 25-hydroxyvitamin D assay to check for possible Vitamin D deficiency.

**AI_C:** RESTATING THE QUESTION: The patient, a 70-year-old woman, has been experiencing slowly progressive fatigue, generalized pain, and muscle aches for a year. She has a medical history of osteoporosis, for which she takes alendronate and calcium supplements. Her latest health checks, including blood counts, hormone concentrations, liver function tests, and others are all normal. Despite these results, she continues to experience the aforementioned symptoms. The question asks for the most appropriate next step in assessing her condition, with the provided options being (A) DEXA scan, (B) electromyography and nerve conduction studies, (C) MRI of the cervical spine, (D) serum antinuclear antibody assay, (E) serum 25-hydroxyvitamin D assay, or (F) no further evaluation. PLANNING: We have to consider several assumptions: one is that her symptoms stem from age; another is that her symptoms are due to her osteoporosis; yet another is that they may be due to another unidentified condition. If her symptoms are age-related, no additional evaluation may be needed. If due to osteoporosis, a DEXA scan might help, focusing on bone density. If due to another condition, additional evaluations would be necessary, possibly looking for an autoimmune condition, nerve disorder, or vitamin deficiency. CLINICAL REASONING: However, her osteoporosis is being treated with alendronate and calcium supplementation, suggesting that her symptoms are likely not due to osteoporosis. Furthermore, absence of point tenderness and normal lab parameters, including erythrocyte sedimentation rate, make inflammatory, infectious, and many systemic diseases less likely. The fact that her symptoms are diffuse and involve generalized pain and fatigue, coupled with the normal results of her other physical examinations and tests, might direct our attention towards a possible deficiency, such as vitamin D deficiency. CONCLUDING: A diagnosis of vitamin D deficiency could explain the generalized musculoskeletal pain and fatigue, as Vitamin D is known to play a role in bone health and muscle function. Several factors put her at risk for vitamin D deficiency, including her age, osteoporosis, and the fact that vitamin D deficiency may be more common in patients with osteoporosis who are taking alendronate. Furthermore, sufficient vitamin D is crucial in calcium homeostasis and bone health, and a deficiency can cause or exacerbate pain and fatigue. RESPONSE: The most appropriate next step in evaluation would be (E) Serum 25-hydroxyvitamin D assay since this will help to evaluate the patient's vitamin D status and could help explain her symptoms. As always, the results should be used in conjunction with her history, physical examination, and other tests before making a final diagnosis.

**AI_E:** RESTATING THE QUESTION: The question entails that a 70-year-old woman is presenting for a regular health check. She reports a year-long history of increasing fatigue, muscular discomfort, and widespread body pain. Notably, she was diagnosed with osteoporosis 5 years ago. Her medication regimen consists of alendronate and calcium supplements. She has normal vital signs, and her physical exam, inclusive of palpation, doesn't reveal anything out of the ordinary expected with the aging process. Her bloodwork is routine, including CBC, serum electrolytes, thyroid hormones, liver functions, and erythrocyte sedimentation rate all registering within reference levels. Recent cancer screenings for breast and colon turned up nothing unusual. The inquiry is asking what evaluation should be next in line for this patient. PLANNING: The patient's sex maybe one of the main contributors of her current distress, as females, particularly postmenopausal women, are at face higher risk of having osteoporosis due to the decline in protective estrogen levels at this stage of life. Given her history of osteoporosis, it's plausible that her existing symptoms stem from an inadequate intake or an impaired metabolism and absorption of essential components for bone health, such as Vitamin D and calcium. It is also known that Vitamin D deficiency can cause nonspecific symptoms such as fatigue and widespread pain. CLINICAL REASONING: Even though her physical exam didn't reveal any localized tenderness or abnormalities, the generalized pain and muscle aches could be manifestations of a systemic problem such as Vitamin D deficiency, considering that Vitamin D plays a crucial part in bone metabolism and muscle function. The other possible investigations, such as DEXA scan, EMG and nerve conduction studies, MRI of the cervical spine, and serum antinuclear antibody assay may not be as pertinent at this time. CONCLUDING: Because of her age, history of osteoporosis, and these chronic nonspecific complaints, it's rational to assess her serum 25-hydroxyvitamin D levels as a next step. This will help us determine if she has an insufficient intake or absorption of Vitamin D, which can affect her bone health and potentially explain her symptoms. RESPONSE: The most appropriate next step in the evaluation of this patient would be (E) Serum 25-hydroxyvitamin D assay.

**AI_D:** (1) RESTATING THE QUESTION: This 70-year-old woman is presenting with symptoms of progressive fatigue, generalized pain, and muscle aches for the last year. Her medical history includes osteoporosis, for which she is taking medication, including alendronate and calcium. Her physical, laboratory, and screening investigations have not shown any obvious abnormalities or reasons for her complaints. The question then is, what could be the best next step for her evaluation out of the options provided? (2) PLANNING: The possible assumptions could be that she is suffering from another systemic condition causing her complaints, or her symptoms may be related to her medication, or to a lack of proper vitamin supplement. Her alendronate use, to treat osteoporosis, could be associated with generalized pain which is often misdiagnosed and may be lacking the necessary supplementation of Vitamin D, crucial for calcium absorption and bone health. (3) CLINICAL REASONING: Although several tests could provide more information about the patient's condition, it is necessary to question each assumption and consider the potential for false positives and unnecessary tests which may not yield useful results. For instance, a DEXA scan could be useful for monitoring her bone density given the history of osteoporosis, but it would not necessarily explain her fatigue and aches, given she is already under treatment for osteoporosis. Similarly, Electromyography and nerve conduction studies could help determine if a neurologic disorder is causing her muscle aches, MRI could evaluate for potential spinal cord or nervous system issues, and a Serum antinuclear antibody assay could determine if she has an autoimmune condition. However, all these tests should have yielded correlating physical examination findings. The test that would provide the most useful information, given her symptoms, age, and risk factors, would be a Serum 25-hydroxyvitamin D assay. The fatigue, muscle aches, and generalized pain could potentially be attributed to low levels of this vitamin, as it is crucial to maintain proper bone health and muscle function. Given her age and medical history of osteoporosis, this could be the most beneficial next step in evaluating her symptoms. (4) CONCLUDING: While a myriad of tests could provide more information about the patient's condition, it is necessary to prioritize what would be the most clinically relevant and beneficial to her. Considering the range of her symptoms, her medical history, the medications she takes, and her age and risk factors, it would make sense to test her serum 25-hydroxyvitamin D levels as this could explain her symptoms and directly assist in her treatment. (5) RESPONSE: Therefore, the most appropriate next step for this 70-year-old woman with progressive fatigue, muscle aches, and generalized pain, would be to conduct a serum 25-hydroxyvitamin D assay test (Option E), given its potential clinical significance in these symptoms and her overall management.

**Facilitator** The consensus among the AI responses is that the most appropriate next step in evaluating the 70-year-old woman's condition is to conduct a Serum 25-hydroxyvitamin D assay (Option E). This conclusion is based on her age, history of osteoporosis, and the nonspecific symptoms she presents, such as fatigue, diffuse muscle aches, and generalized pain. Despite her current treatment for osteoporosis with alendronate and calcium supplements, these symptoms persist, suggesting that they may not be directly related to her osteoporosis. The AI responses also highlight that while other tests could provide additional information, they may not be as relevant or beneficial given the patient's current symptoms and medical history. For instance, a DEXA scan might be useful for monitoring her bone density, but it would not necessarily explain her fatigue and aches. Similarly, electromyography and nerve conduction studies, an MRI of the cervical spine, or a serum antinuclear antibody assay could help identify a neurological or autoimmune condition, but these are less likely given her normal physical examination and lab results. The reasoning behind the suggested Vitamin D test is that Vitamin D plays a crucial role in bone health and muscle function, and a deficiency can cause or exacerbate symptoms like fatigue, muscle aches, and generalized pain. Given the patient's age and medical history, she is at a higher risk for Vitamin D deficiency due to factors such as decreased dietary intake, reduced sunlight exposure, and diminished capacity for cutaneous synthesis of Vitamin D. Therefore, a Serum 25-hydroxyvitamin D assay could provide valuable information about her Vitamin D levels and potentially explain her symptoms.
